# Rapid Infancy Weight Gain and 7- to 9-year Childhood Obesity Risk: A Prospective Cohort Study in Rural Western China

**DOI:** 10.1097/MD.0000000000003425

**Published:** 2016-04-22

**Authors:** Jing Zhou, Shaonong Dang, Lingxia Zeng, Wenlong Gao, Duolao Wang, Qiang Li, Wenhui Jiang, Leilei Pei, Chao Li, Hong Yan

**Affiliations:** From the Department of Epidemiology and Biostatistics, School of Public Health, Health Science Center, Xi’an Jiaotong University, Xi’an, Shaanxi, P.R. China (JZ, SD, LZ, QL, LP, CL, HY); Department of Epidemiology and Health Statistics, School of Public Health, Lanzhou University, Lanzhou, Gansu, P.R. China (WG); Liverpool School of Tropical Medicine, University of Liverpool, Liverpool, UK (DW); Faculty of Nursing, Health Science Center, Xi’an Jiaotong University, Xi’an, Shaanxi, P.R. China (WJ); and Nutrition and Food Safety Engineering Research Center of Shaanxi Province, Xi’an, Shaanxi, P.R. China (HY).

## Abstract

Obesity is increasing in developing countries. This study aimed to identify the association between rapid infancy weight gain and obesity risk among early school-age children.

A total of 581 singletons (349 boys, 232 girls) whose mothers participated in an antenatal multiple micronutrient supplement trial in rural western China were followed from birth to between 7 and 9 years of age. Height and weight were measured at birth, 1.5 years, and between 7 and 9 years. At the 7- to 9-year time point, body composition was determined using bioelectrical impedance analysis. Multilevel mixed analysis was used to test the associations between rapid weight gain in infancy (from birth to age 1.5 years) and body size and composition or overweight/obesity among early school-age children.

Overall, 31.2% (181 of 581) of the infants showed a weight-for-age Z score gain greater than 0.67 between birth and 1.5 years, indicating rapid weight gain. Approximately 5.7% (33 of 579) of the subjects were overweight (BMI-for-age Z scores [BAZ] >1 and ≤2) or obese (BAZ >2). Rapid infancy weight gain was associated with a higher BAZ (*P* < 0.001), mid-upper arm circumferences (*P* < 0.001), percentage body fat (*P* < 0.001), and fat mass index (*P* < 0.001) at 7 to 9 years of age after adjusting for biological and social economic factors, genetic factors, and perinatal and postnatal factors. These associations appeared to be independent of gender, economic status at early school age, and maternal nutritional status at enrollment. Rapid growers may have approximately 3 times the risk of being overweight/obese during the early school-age years (odds ratio = 2.94, 95% CI: 1.17–7.43, *P* = 0.022).

Rapid infancy weight gain is a risk factor for being overweight/obesity among early school-age children in rural western China. We propose that social and biological determinants, such as economic status, physical activity, and feeding practice, should be targeted to prevent obesity.

## INTRODUCTION

The prevalence of childhood obesity is increasing in a similar manner in many low- and middle-income countries in relation to increasing affluence.^1^ In 2009 in China, 18.8% of 7 to 12-year-old children from urban areas were overweight and 8.0% were obese, whereas 8.9% of 7 to 12-year-old children from rural areas were overweight and 5.3% were obese.^2^ Childhood obesity has been linked to various adverse physical, psychological, and social outcomes, including type 2 diabetes, cardiovascular disease, cancer, depression, low self-esteem, and diminished quality of life.^3–5^ Evidence indicates that once obesity is established, it is difficult to reverse through interventions,^6^ and it tends to persist into adulthood.^7^ Therefore, in addition to treatment, the prevention of childhood obesity has become a global public health priority.

To prevent obesity, both the risk factors and the critical periods for the development of obesity must be clearly identified. In a large birth cohort study,^8^ 30.7% of infants showed catch-up growth between zero and 2 years, and these children had greater body mass index (BMI), percentage fat mass, and waist circumference at 5 years. Systematic reviews^9–11^ have consistently shown that rapid infancy weight gain is associated with an increased risk of obesity in later life. However, most studies included subjects born before the 21st century.^8,12,13^ It is unclear whether the association between infancy weight gain and later obesity is still valid today. The objective of our study was to evaluate the association between rapid infancy weight gain and later body size and composition or overweight/obesity in a 2004 Chinese birth cohort study.

## METHODS

### Study Population

This study is a prospective study of growth in the offspring of mothers who had participated in a cluster-randomized, double-blinded, controlled trial (ISRCTN08850194).^14^ The trial was conducted in 2 poor rural counties in Shaanxi Province in northwest China from August, 2002 to January, 2006. The 2 counties have experienced rapid economic growth over the last decade because of a thriving coal mine industry.

The details of the trial have been described elsewhere.^14^ Briefly, pregnant women in the same village received the same daily antenatal micronutrient supplementation from study enrollment until delivery. Postnatal growth was measured at birth, every 3 to 12 months of age, every 6 months between 12 and 30 months of age, and at 7 to 9 years. In total, 5828 mothers were invited to participate in the study. They were followed during and after pregnancies; 1388 live singletons born in 2004 (the middle year of the total 3.5 years of recruitment) completed the follow-up study from birth to 30 months. A total of 562 children were excluded: 5 died, 407 moved out of the study area (and were therefore excluded for having a different socioeconomic profile), and 150 were missing birth weight data or weight data at 1.5 years. Among the 826 remaining children, 41 refused to participate, and 204 were unknown locations. Therefore, the final sample consisted of 581 children (349 boys and 232 girls), representing 70.3% of the original 826 eligible participants. Ethical approval was obtained from the Science and Research Ethics Committee of Xi’an Jiaotong University. In each case, a parent provided signed consent, and verbal assent was obtained from each child.

### Anthropometric Measurements

Birth weight and length were measured by hospital nursing staff within 1 hour of delivery. Birth weight was measured with an electronic scale (BD-585, Tanita Corporation, Dongguan, Guangdong Province, China) with precision to the nearest 10 g. Birth length was measured to the nearest 0.1 cm using a portable measuring board with fixed head piece. Weight and length at around 1.5 years were available from routinely infant home visit performed by trained research fieldworkers and nurses and were documented in the infant's personal health record. Weight was measured to the nearest 10 g with the electronic scale, excluding the weight of children's clothes. Recumbent length was measured to the nearest 0.1 cm on the length board. At early school-age, weight, standing height, mid-upper arm circumference (MUAC), and body composition were assessed by trained investigators. The subjects were dressed in underwear only and were barefoot. Weight and body composition was evaluated using bioelectrical impedance (BC-420, Tanita Corporation, Tokyo, Japan). Standing height was measured to the nearest 0.1 cm using a steel strip stadiometer (SZG-210, Shanghai JWFU Medical Apparatus Corporation, Shanghai, China). In our cohort, the coefficient of variation for weight at birth, 1.5 years, and early school-age were 13.2%, 10.2%, and 15.7%, respectively, and for length/height were 5.2%, 3.8%, and 4.4%, respectively. MUAC was measured on the right arm at the level of the upper arm midpoint mark to the nearest 1 mm using a nonelastic tape (MagicFit factory, Beijing, China).

### Outcomes of Interest and Exposure Variable

BMI was calculated as the body weight in kilograms divided by the height in meters squared. Sex- and gestational-age adjusted birth weight and length Z scores were calculated according to global standards^15^ and computed using the birth size Z scores R script.^16^ Sex- and age-adjusted Z scores for weight, height, and BMI at 1.5 years and early school-age were calculated using the World Health Organization (WHO) reference-data growth charts.^17,18^ Rapid infancy weight gain was defined as a gain in Z score greater than 0.67 for weight between birth and 1.5 years.^8^ BMI-for-age Z scores (BAZ), MUAC, percentage body fat (PBF), and fat mass index (FMI) were used as markers of overweight/obesity. Children with a BAZ between +1 and +2 were classified as overweight, and those with a BAZ above +2 were classified as obese.^19^ PBF was FM divided by total body mass times 100. FMI was FM in kilograms divided by the height in meters squared, which was calculated to adjust the body composition for height.

### Covariates

For all analyses, we included all corresponding covariates in the models based on their associations with rapid infancy weight gain or with childhood body size and composition based on previous studies.^20–27^ Parental height and weight were measured at early school-age according to the standard procedures. Information on other variables were collected by face-to-face interview using self-designed structured encoded questionnaire. All of these data were recorded during the original trial and the follow-up study. We adopted the hierarchical approach proposed by Victora et al^28^ to develop a conceptual framework, which grouped all covariates into 3 domains (biological and socioeconomic factors, genetic factors, and perinatal and postnatal factors). Biological and socioeconomic factors included: gender (male, female), age, birth weight, gestational age, family economic status at early school age (poorer, wealthier), parental educational level (primary or below, above primary), and parental occupation (farmers or not). Genetic factors included: parental height (below 10th percentiles among study objectives or not) and parental BMI (above 25 kg/m^2^ or not). Perinatal and postnatal factors included: parity (primipara or not), maternal malnutrition at enrollment (yes or no), antenatal micronutrient supplementation (folic acid, iron + folic acid, or multiple micronutrients), numbers of supplementation consumed (<180, ≥ 180), infant feeding method (appropriate or not), activity level (appropriate or not), and medical history (serious diseases during infant or not). Gestational age was computed as the number of completed weeks of gestation from the date of the mother's last menstrual period to delivery. Family economic status was based on an assets index divided into dichotomies to indicate the poorer and wealthier households. The mothers were considered as primipara if they reported “no previous pregnancy” or “no children living in the household” at enrollment, depending on which question was asked at that time. Maternal MUAC at enrollment was used as an indicator of short-term nutritional status, and maternal malnutrition was defined as women with MUAC below 23.5 cm.^29^ Appropriate feeding was defined as exclusive breastfeeding until 6 months and the appropriate introduction of complementary food (i.e., after 6 months and with high-quality protein provided at least once per week). Providers chose one of 6 categories (0, 1–30, 31–60, 61–120, 121–180, >180 minutes) in response to a question “How many minutes did the infant in your care spend on outdoor activity per day.” We dichotomized their responses to determine whether appropriate (>60 minutes/day) or not.

### Statistical Analysis

The data were checked manually for completeness and were double-entered into EpiData version 3.1 (EpiData Association, Denmark). We performed range and logic checks of data to ensure the accuracy. Initially, mean ± standard deviation (SD) or median with interquartile range were used to describe normally or abnormally distributed continuous variables, respectively. Count and proportions were used to describe categorical variables. We compared baseline characteristics between the follow-up group and the lost-to-follow-up group, and also we compared the characteristics between subjects experiencing rapid infant weight gain or not. Continuous data were analyzed with *t* tests (normally distributed data) or Mann–Whitney *U* test (abnormally distributed data), and categorical data were analyzed with χ^2^ tests. Because of the hierarchical structure of the data, we adopted 3-level mixed analysis to estimate the effects of rapid infancy weight gain on markers of overweight/obesity at early school age with township to level 3, village to level 2, and individual to level 1; the outcomes included the BAZ, MUAC, PBF, and FMI (continuous variables). Following the conceptual hierarchical framework, sequential models were constructed and tested as follows: with no adjustment (model 0); with adjustments for biological and socioeconomic factors (model 1); with adjustments for all variables in model 1 plus genetic factors (model 2); and with adjustments for all variables in model 2 plus perinatal and postnatal factors (model 3). Another 3-level mixed analysis was used to test the effects of rapid infancy weight gain on the risk of overweight/obesity (dichotomous variable) at early school age, with the same adjustments that were used in models 0 to 3. In all these models, the presence or absence of rapid infancy weight gain and potential confounders were treated as fixed effects, whereas township, village, and individual were treated as random effects. To test the impact of those lost to follow-up, an additional analysis was conducted after imputation of missing values using expectation-maximization. The main results were consistent between analysis on those follow-up and imputed analysis. Furthermore, we performed sensitivity analysis excluding preterm (gestational age at birth < 37 weeks) born children. We also performed sensitivity analysis to examine the potential modifying effects of gender, family economic status at early school age, and maternal nutritional status at enrollment on the associations between rapid infancy weight gain and BAZ, MUAC, PBF, and FMI. All reported *P* values were 2-tailed, and the level of significance was set at 0.05. The analyses were performed using SPSS version 20.0 (International Business Machines Corporation, Armonk, NY).

## RESULTS

### Cohort Characteristics

**FIGURE 1:**
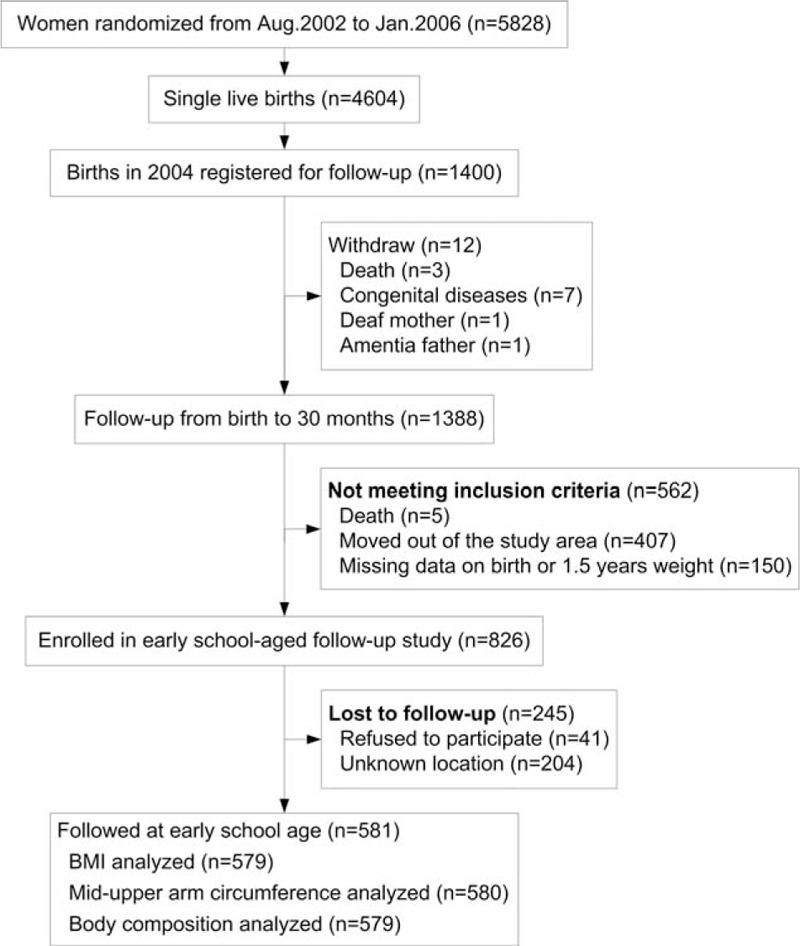
Participant flow chart.

Figure 1 shows the flow chart of participants from the original trial through the follow-up study. Among the school-age anthropometric measures, BMI was missing for 2 participants, MUAC was missing for 1 participant, and body composition was missing for 2 participants. Baseline characteristics were similar between the participants who were lost to follow-up and those who completed the study (Table 1). Of the 581 children who were followed, the mean age was 8.29 years (±0.51 years). In all, 31.2% children demonstrated rapid weight gain between birth and 1.5 years of age, and the total prevalence of overweight/obesity was 5.7%.

**TABLE 1:**
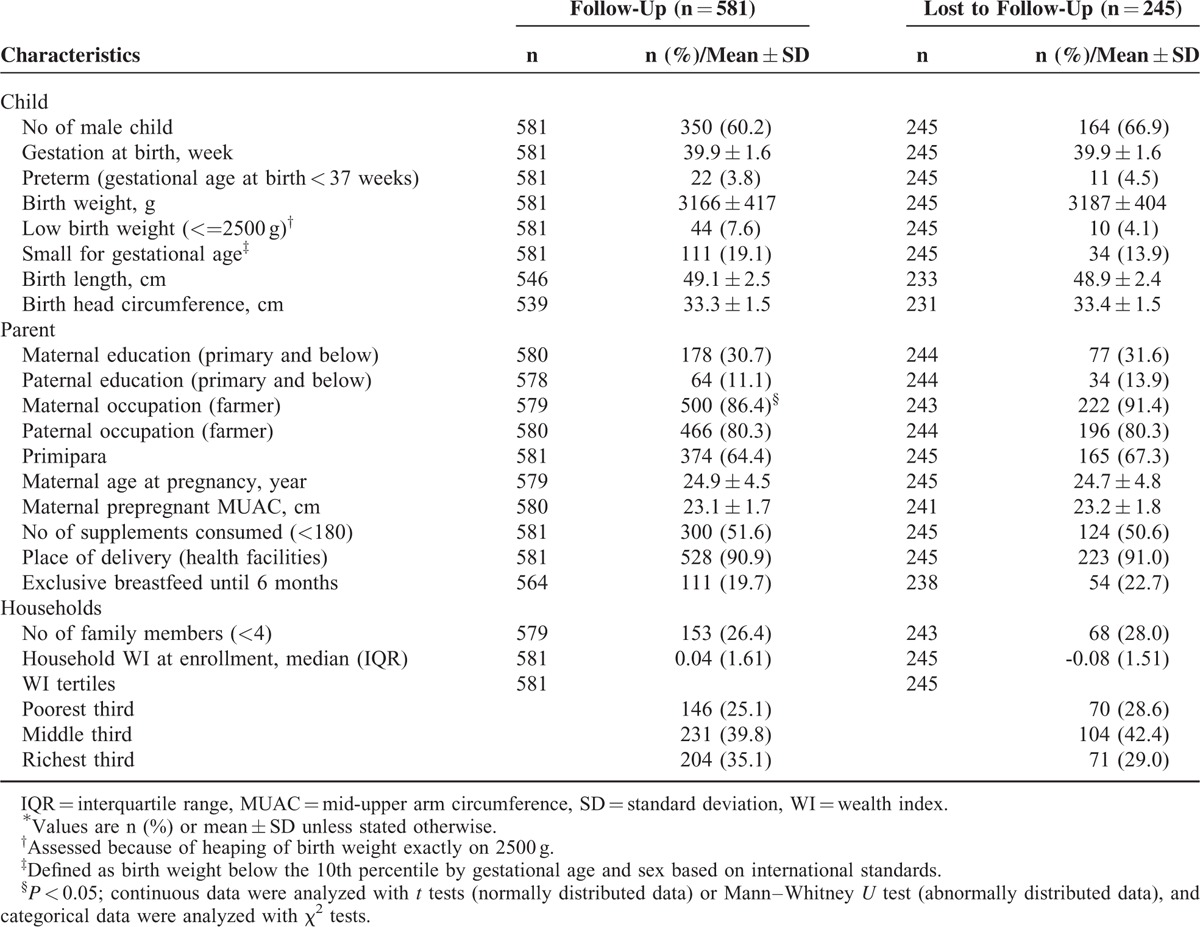
Baseline Characteristics of Follow-Up and Lost to Follow-Up Participants^∗^

### Body Size, Body Composition, and Overweight/Obesity

The average weight and length/height Z scores at different period of ages were all below the WHO growth reference 2007 (Table 2). At the early school age, the girls had a significantly higher FM (*P* = 0.009), PBF (*P* < 0.001), and FMI (*P* < 0.001) than the boys; however, the total prevalence of overweight/obesity was significantly higher among the boys than that of the girls (*P* = 0.009, Table 2).

**TABLE 2:**
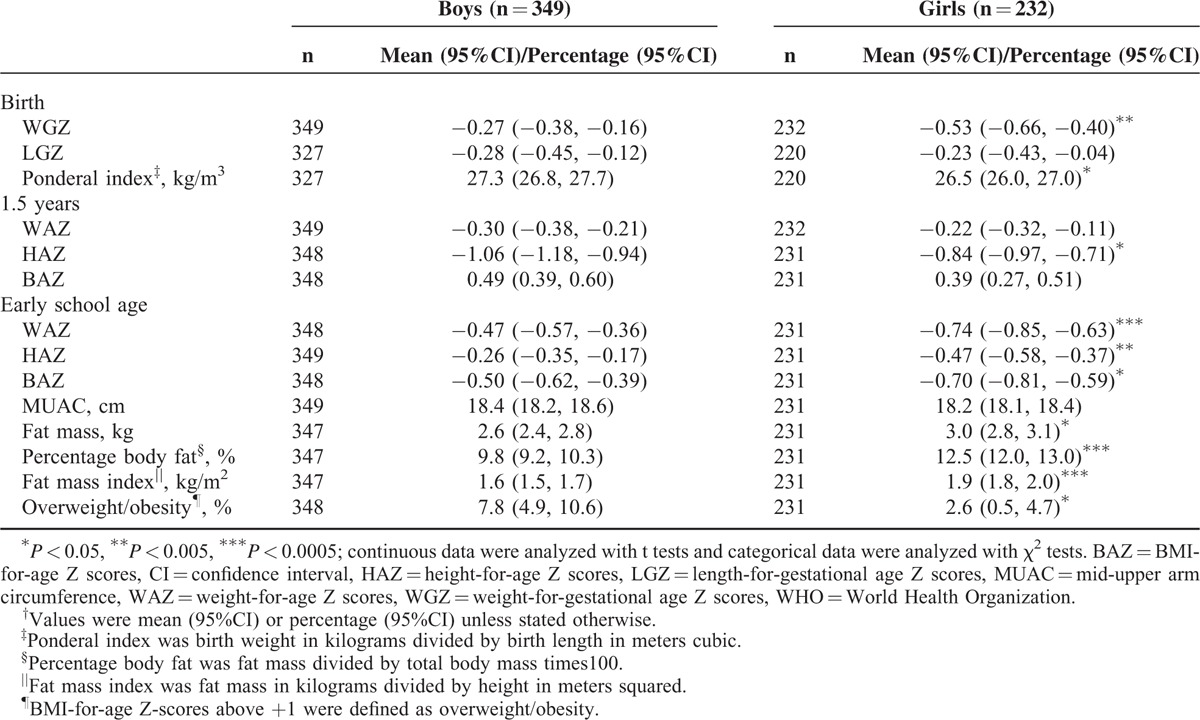
WHO Standard Z Scores for Size at Birth, 1.5 years, and Early School Age^†^

### The Characteristics Between Rapid Growers and Nonrapid Growers

As compared with nonrapid growers, rapid growers had mothers who were higher educated and ate less number of supplementations during pregnancy (Table 3). Moreover, their fathers were more likely to be overweight. As compared with nonrapid growers, rapid growers had a lower birth weight, more frequently being first-born, and less often exclusively breastfed until 6 months.

**TABLE 3:**
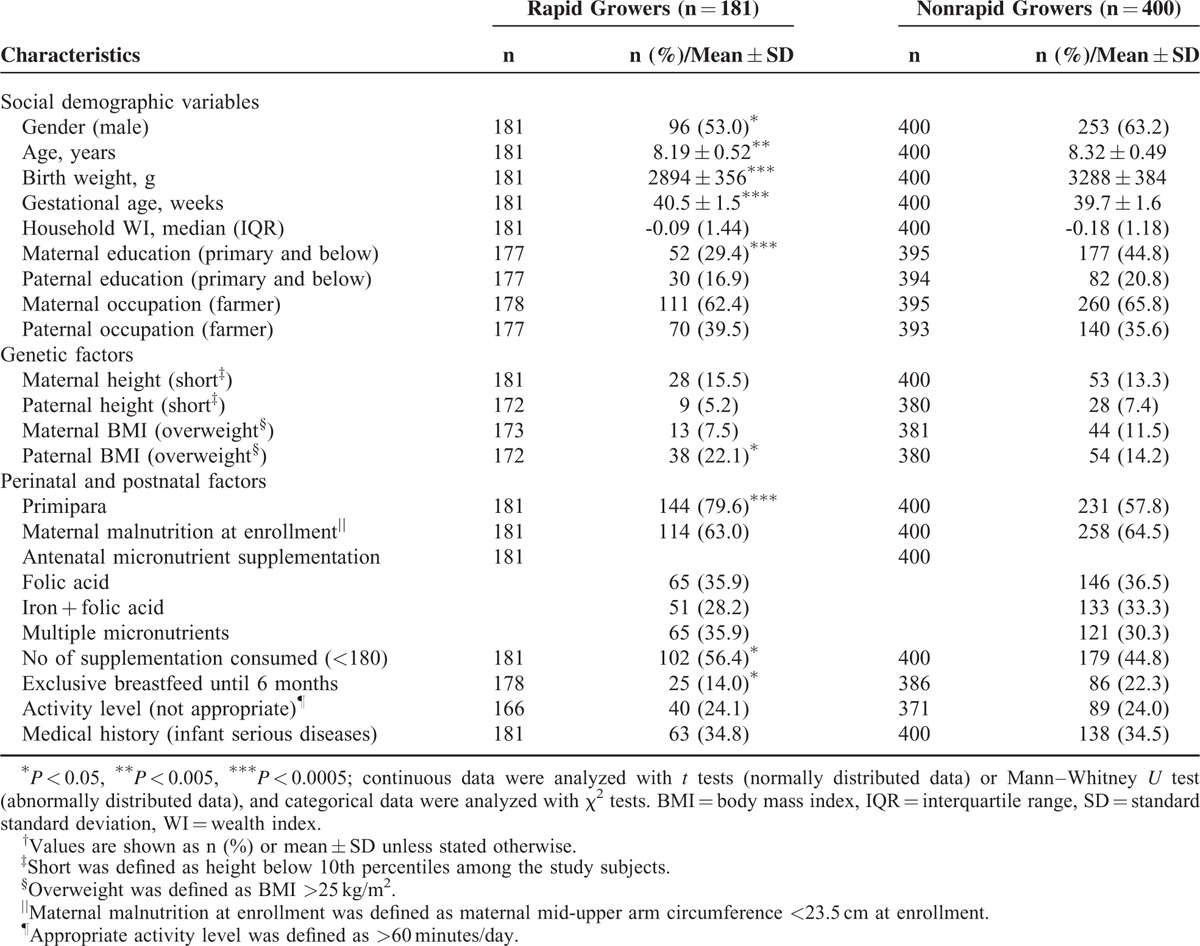
Characteristics Between Subjects Experiencing Rapid Infancy Weight Gain or Not^†^

### The Associations Between Rapid Infancy Weight Gain and Body Size, Body Composition, and Overweight/Obesity at Early School Age

**FIGURE 2:**
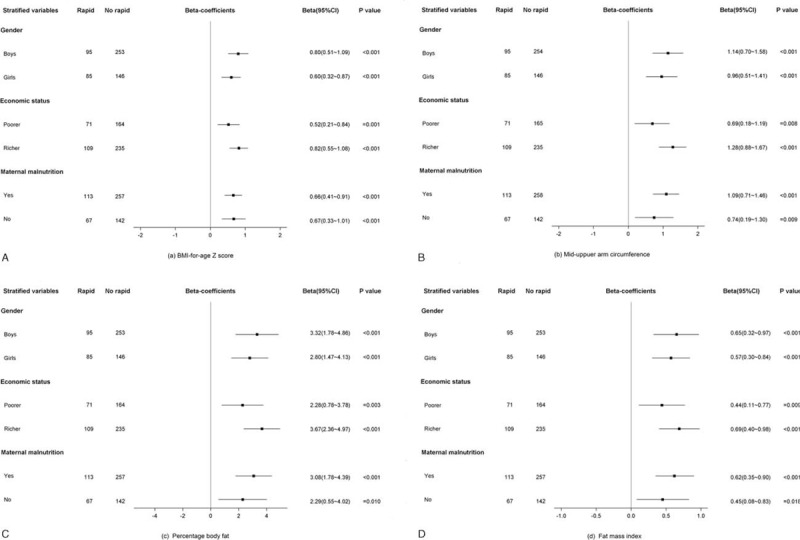
(A) The effect of rapid infancy weight gain on BMI-for-age Z score at early school age by gender, economic status at early school age, and maternal nutritional status at enrollment. (B) The effect of rapid infancy weight gain on mid-upper arm circumference at early school age by gender, economic status at early school age, and maternal nutritional status at enrollment. (C) The effect of rapid infancy weight gain on percentage body fat at early school age by gender, economic status at early school age, and maternal nutritional status at enrollment. (D) The effect of rapid infancy weight gain on fat mass index at early school age by gender, economic status at early school age, and maternal nutritional status at enrollment. 95% CI: 95% confidence interval; reference category: no rapid weight gain between birth and 1.5 years; three-level mixed analysis was used, with adjustments for gender, age, birth weight, gestational age, economic status at early school age, parental educational level, occupation, height, BMI, maternal malnutrition at enrollment, antenatal micronutrient supplementation, birth order, infant feeding method, activity level, and medical history. BAZ = body mass index-for-age Z scores, BMI = body mass index, CI = confidence interval, FMI = fat mass index, MUAC = mid-upper arm circumference, PBF = percentage body fat.

In the unadjusted univariate analysis (Table 4), the rapid growers showed significantly greater BAZ, MUAC, PBF, and FMI and had an increased risk of overweight/obesity compared with the nonrapid growers at early school age. After adjusting for biological and socioeconomic covariates, the mean differences and odds ratio were all strengthened (model 1); these estimates and odds ratio were only modestly attenuated after additional adjustment for genetic (model 2) and perinatal and postnatal covariates (model 3). Results of sensitivity analysis excluding preterm born children (data not shown) showed similar associations. Regardless of gender, economic status at early school age, or maternal nutrition status at enrollment, the positive associations between rapid infancy weight gain and the early school-age BAZ (Figure 2A), MUAC (Figure 2B), PBF (Figure 2C), and FMI (Figure 2D) persisted.

**TABLE 4:**
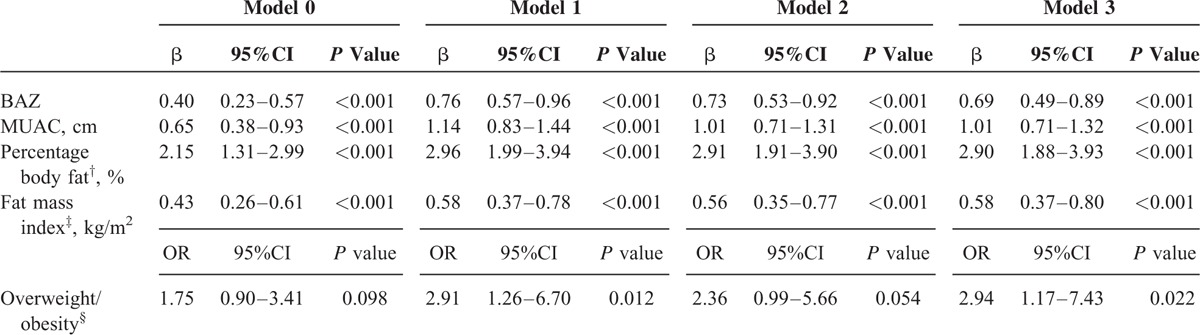
Multilevel Mixed Analysis of the Effect of Rapid Infancy Weight Gain on the Markers of Overweight/Obesity at Early School Age^∗^

## DISCUSSION

### Major Findings and Their Significance

In this prospective cohort study, we evaluated the associations between rapid infancy weight gain with markers of overweight/obesity (BAZ, MUAC, PBF, and FMI) in childhood among children born in the 21st century. Approximately one-third of the children in this cohort grew rapidly between birth and 1.5 years of age. Our results showed that rapid infancy weight gain was significantly associated with an increase in BAZ, MUAC, PBF, and FMI of the order of 0.5 SD at early school age. Importantly, rapid infancy weight gain increased a child's risk of being overweight/obesity at early school age by almost 3 times after adjustments for other confounders.

### Interpretation and Comparison With Other Studies

The effect of early rapid growth on later obesity risk that was observed in this cohort expands the findings of some previous reviews.^9–11^ Previous studies conducted in developed countries have shown that children with rapid infancy weight gain tend to have a higher BMI and are at increased risk of being overweight during childhood,^8,30^ adolescence,^31^ and adulthood.^13,32^ A prospective cohort study^8^ of 848 full-term children suggested that children with accelerated growth between birth and 2 years of age (an increase in weight-for-age Z scores > 0.67) had a greater BMI at 5 years of age. In addition, another study^33^ of 136,971 regularly followed children in south China found that rapid growers had a higher risk of being overweight (BAZ >  +2) at 4 to 5 years of age than the others. Note that BMI is a marker of general change in body size, while body composition can reflect a detailed change in body components. In our study, children with rapid infancy weight gain had a higher PBF and a higher FMI at early school age. These findings align with those of other studies showing that rapid early postnatal growth may lead to higher levels of total body fat in childhood.^8,34,35^ A prospective study of 233 white adults suggested that rapid infancy weight gain was associated with an elevated risk of obesity, higher total body fat, PBF, and visceral adipose tissue mass.^32^ However, in our study, we could not explore the effect of rapid infancy weight gain on total body fat deposition (subcutaneous vs visceral), which exerts distinct effects on metabolic syndrome, diabetes risk, and circulating adipokine levels.^36^

### Social Determinants of Obesity

An evidence from 70 countries found that obesity was positively associated with GDP among individuals with lower levels of education.^37^ Our study was conducted in the backward, rural region, which had low levels of education. We could see a great influence of economic status on the associations between rapid infancy weight gain and body size, body composition. Not only social inequalities but also children themselves should be responsible for their nutritional status. A study among an international sample of 9 to 11-year-old children indicated that greater physical activity was associated with lower odds of obesity.^38^ Outdoor activity during infant was within the range of controlled confounding in our study. Recent evidence suggested that fathers had a unique and key role in shaping their children's physical activity behaviors.^39,40^ More researches were needed to explore the efficient behavioral interventions that could improve the living style and health of children.

### Biological Determinants of Obesity

Our results support etiologic hypotheses concerning critical periods or pathways for obesity development. Identifying the mechanisms that underlie the association between rapid infancy weight gain and later obesity is likely to be important for understanding how to prevent obesity throughout life. Some hypotheses about possible mechanisms have already been suggested based on previous observational studies and experimental models.^41,42^ The evidence for a lower growth rate in breastfed children compared to formula fed children has been rather consistent.^43^ Formula has higher protein and energy density than human milk, which stimulates the secretion of insulin-like growth factor 1.^44^ Insulin-like growth factor 1 improves protein synthesis and cell proliferation, and then accelerates growth and increases muscle mass and adipose tissue.^45^ In our study, whether being exclusively breastfed until 6 months was associated with rapid growth, which may suggest that rapid infancy weight gain could be associated with a diet-induced altered metabolic state in infancy. Rapid infancy weight gain has also been associated with changes in appetite regulating hormones (e.g., leptin, adiponectin, and ghrelin),^46^ which result in increased appetite.^47^ A cohort of term appropriate-for-gestational-age infants^46^ showed that an increase in sex- and age-independent SD scores for weight during infancy was associated with lower concentrations of adiponectin, which may predict future insulin resistance.^48^ Moreover, a birth cohort study found an association between higher androgen metabolite excretion and faster postnatal weight gain among girls.^49^ It could be hypothesized that rapid infancy weight gain programs overactivity of the hypothalamic-pituitary-adrenal axis, which result in central obesity and insulin resistance.^49^ A prospective observational study reported that maternal prepregnant BMI was positively associated with infant weight gain from birth to 1 year.^50^ Therefore, the mechanisms linking rapid infancy weight gain and childhood obesity may also include transgenerational factors, such as genetic or epigenetic factors, or familial behaviors. Prospective studies that carefully characterize childhood growth, body composition, and genetic, endocrine, metabolic, nutritional, and social factors would advance our understanding of these complex associations.

### Study Strengths and Limitations

The main strength of this analysis is the long-term follow-up. Prospective data for potential confounders and growth were available in this study, which decreased the risk of recall bias or unreliable measurements. As with any observational study, caution should be exercised when inferring causality based on the findings. However, our findings were consistent before and after adjustments for potential confounding by most known variables that are plausibly associated with infancy and childhood growth. The associations were still consistent in the sensitivity analysis.

Our study also has some limitations, such as the high sample attrition rate. Among the children with available weight information at birth and 1.5 years of age who were still living in the study areas, 70.3% participated in the follow-up measurements at early school age, 99.7% of these children also participated in the body composition assessments. Mothers who were farmers were less likely to be excluded from the current analyses than included (91.4% vs 86.4%, *P* < 0.05). It is difficult to speculate whether this difference significantly affected the observed associations, but we consider this possibility unlikely. We assumed that the sample attrition followed a missing-at-random pattern; therefore, the results of the present study can be generalized to the original sample. Although our study was conducted within the context of a clinical trial for antenatal micronutrient supplementation, we did not find any evidence of modified associations within the trial intervention arm. The relatively small sample size reduced the power to detect differences in the risk of being overweight/obesity between rapid growers and nonrapid growers. We need to be prudent in making conclusions about the association between rapid growth and risk for overweight/obesity. However, we had a more than 99% power to detect differences in markers of overweight/obesity of the order of 0.5 SD between groups. Other study limitations included a lack of information about body composition or other biochemical markers of chronic disease risk during infancy. It is possible that changes in infant adiposity, body composition, and even biochemical markers might predict future obesity risk more clearly than simple weight gain does. We are not aware of any long-term studies with such detailed characterization during infancy. However, birth weight and infant weight are easily measured, and such information is widely available; therefore, such information could be easily used to screen large populations. We used the international standards developed by the WHO to calculate the Z scores and to identify rapid weight gain and overweight/obesity, thereby enabling more meaningful comparisons across different cultures and settings. Besides, the most frequent definition for rapid growth was a Z-score change > 0.67 in weight-for-age,^51^ which may be readily clinically interpreted as crossing upward by at least 1 percentile band on standard growth chart. Finally, physical activity during school age was not within the range of controlled confounding in our study. As physical activity was assessed by a self-reported questionnaire, which may be confounded by recall bias. Moreover, we only assessed the frequency of physical activity during school age, but not included the information about intensity and volume. Although we performed an extensive adjustment for a large number of potential confounders, unobserved confounding factors might still occur among the observed associations, as is possible in any observational study.

### Clinical Implications and Conclusions

This study concluded that rapid infancy weight gain is a risk factor for obesity at early school age among children born in the 21st century in rural western China. Rapid infancy weight gain is a risk factor for childhood obesity. Using growth charts to identify at-risk infants could reduce levels of child overweight and obesity by enabling health care professionals to target prevention more effectively. Further research needs to evaluate the clinical validity and feasibility of this risk factor. Further exploration of genetic and postnatal environmental factors that might influence rapid postnatal growth could uncover modifiable mechanisms that underlie the association between growth in infancy and risks for later obesity. Longer follow-up studies of this study population and other populations are needed to assess whether these associations persist and other associations develop in adolescence and adulthood.
